# A rare synchronous occurrence: ovarian and renal primary tumors detected simultaneously: a rare case report

**DOI:** 10.1097/MS9.0000000000003214

**Published:** 2025-04-10

**Authors:** Mouna Baddoura, Ahmad Idelbi, Reem Salman, Souheb Al-Mahasna

**Affiliations:** a Faculty of Medicine, Damascus University, Damascus, Syrian Arab Republic; b Department of General Surgery, Al-Mouwasat University Hospital, Damascus, Syrian Arab Republic

**Keywords:** case report, kidney, ovarian cancer, ovary, renal cell carcinoma, synchronous tumors

## Abstract

**Introduction and importance:**

Multiple primary malignancies (MPMs) are separate malignant tumors in the same patient that are not caused by metastasis or recurrence; they represent between 1.84% and 3.9% of all malignancies. Synchronous MPMs, where the tumors are detected at the same time, are even rarer, with a rate of about 0.7%. Moreover, synchronous primary neoplasms involving both the kidneys and ovaries are extremely rare and have been reported in only a few cases in medical literature.

**Presentation:**

We present the first case to be reported in Syria as multiple primary tumors of a 62-year-old woman who presented with heaviness in her lower abdomen and right hypochondrium and was found to have synchronous primary tumors, left renal clear cell carcinoma and right ovarian papillary serous cystadenocarcinoma based on computed tomography-scan and histopathology. The patient underwent left radical nephrectomy and resection of the right ovary and will receive adjuvant chemotherapy, with regular follow-up recommended to monitor for any signs of recurrence or metastasis.

**Discussion:**

Based on our literature review, the co-occurrence of ovarian tumor with renal cell carcinoma is uncommon. Distinguishing between metastatic tumors and simultaneous primary tumors presents a significant diagnostic challenge. Achieving a precise diagnosis requires the observation of markedly different histologies to confirm the presence of concurrent primary tumors.

**Conclusion:**

This case serves as a reminder that when a patient presents with multiple masses, it is important to consider the possibility that they could be separate primary tumors despite their rarity.

## Introduction

Multiple primary malignancies (MPMs) refer to the presence of two or more distinct malignant tumors in an individual that are not a result of recurrence or metastasis^[^1^]^. MPMs in one patient represent between 1.84% and 3.9% of all malignancies^[^2^]^. According to a literature review which included 1 104 269 cancer patients in 2003, the prevalence of MPMs was between 0.73% and 11.7%^[^3^]^. However, the diagnosis of MPMs is increasing in frequency nowadays based on the combination of many factors (diagnosis, treatment, and demographics)^[^1,4^]^. The mechanism of MPMs is still unknown^[^1^]^. The stage of each tumor, the pathological type, and the patient’s general condition are effective factors in treatment, with priority given to the most considerable tumor^[^1^]^. Furthermore, synchronous MPMs are less common, with an incidence of about 0.7%^[^2,5^]^. The most common sites for synchronous primary tumors are the genitourinary and gastrointestinal systems, with breast cancer also frequently involved in combination with either of the previously mentioned systems^[^2^]^. Moreover, kidney and ovary synchronous primary tumors are very rare, with very few reported cases^[^2^]^. Hence, we report a case of a 62-year-old woman who was admitted to our university hospital and then was diagnosed with two primary malignancies, left renal clear cell carcinoma and right ovarian papillary serous cystadenocarcinoma who underwent surgical resection and will receive adjuvant chemotherapy. This case report aims to highlight the diagnostic and management challenges associated with synchronous primary renal and ovarian tumors and to review the existing literature on this rare presentation.

**HIGHLIGHTS**

- Synchronous multiple primary malignancies are tumors that are detected at the same time are extremely rare with a rate of about 0.7%.
- Synchronous primary tumors involving both the kidneys and ovaries are even rarer.
- Distinguishing between coexistent double cancers and metastasis can be challenging before surgery.
- Doctors have to find a therapy suitable for both malignancies without increased toxicity, pharmacological interactions, or negative impact on the overall impact.

## Case presentation

**Figure 1.**
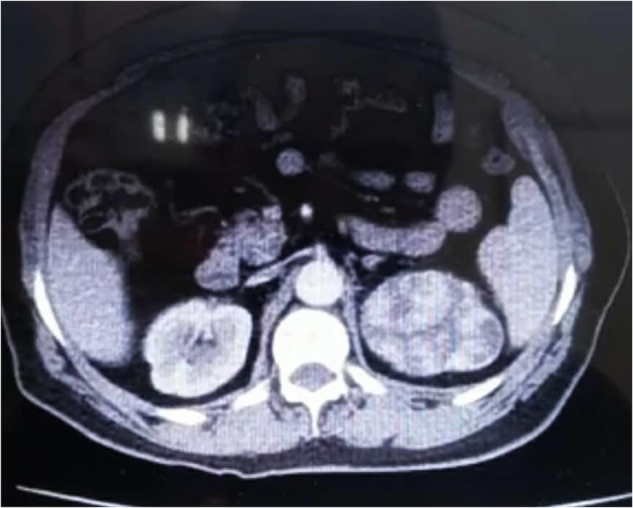
Figure shows 8 × 6 × 7 cm heterogeneous mass at the left kidney.

**Figure 2.**
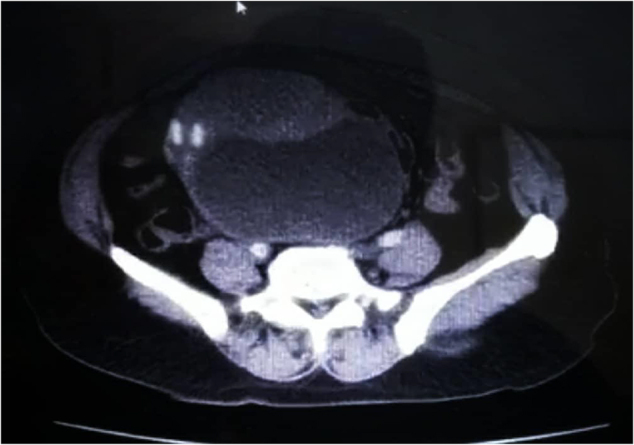
Figure shows complex adnexal solid and cystic mass measuring 11 cm × 11 cm × 6 cm in the pelvis.

**Figure 3.**
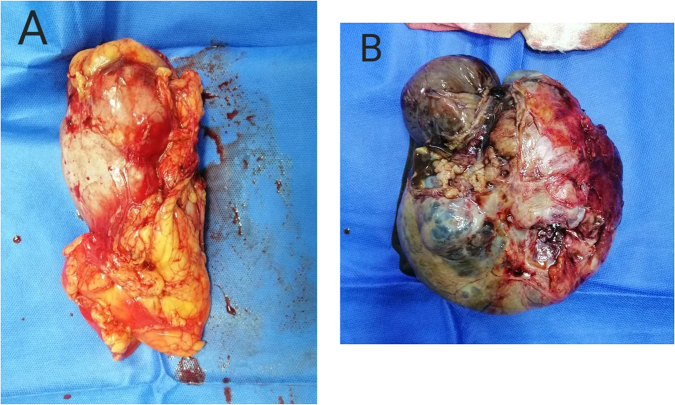
The left kidney (A) and the right ovary (B) after surgery and resection.

A 62-year-old woman presented to our university hospital with complaints of heaviness in the lower abdomen and right hypochondrium for about a month with an ultrasound examination done from outside, which showed heterogeneous hypoechogenic 12 cm tissue mass at the right ovary occupying the right hypochondrium and a 6 cm mass at the upper pole of the left kidney. She is a non-smoker with a history of hypertension for 8 years, managed with amlodipine and atenolol, and had a hysterectomy 13 years ago due to a benign fibroid. There was no record of chronic diseases or cancer in her family history. The patient upon admission was alert, responsive, and oriented; all vital signs were normal. She was further advised for computed tomography (CT) with intravenous contrast, which showed a heterogeneous mass at the left kidney measuring 8 cm × 6 cm × 7 cm (Fig. 1) and a complex adnexal solid and cystic mass measuring 11 cm × 11 cm × 6 cm in the pelvis (Fig. 2). Then, she underwent an open surgery for radical left nephrectomy and bilateral salpingo-oophorectomy to remove both of your ovaries and fallopian tubes (Fig. 3). There were no complications during or after surgery and the patient did not receive blood transfusions.

The macroscopic examination of the resected organs upon the cut section revealed a well confined, encapsulated tumor with hemorrhage, necrosis, and cysts within the upper pole of the kidney and mass with cysts and vegetations on the inner and outer surface of the ovary. Postoperative CT scan didn’t show metastasis.

**Figure 4.**
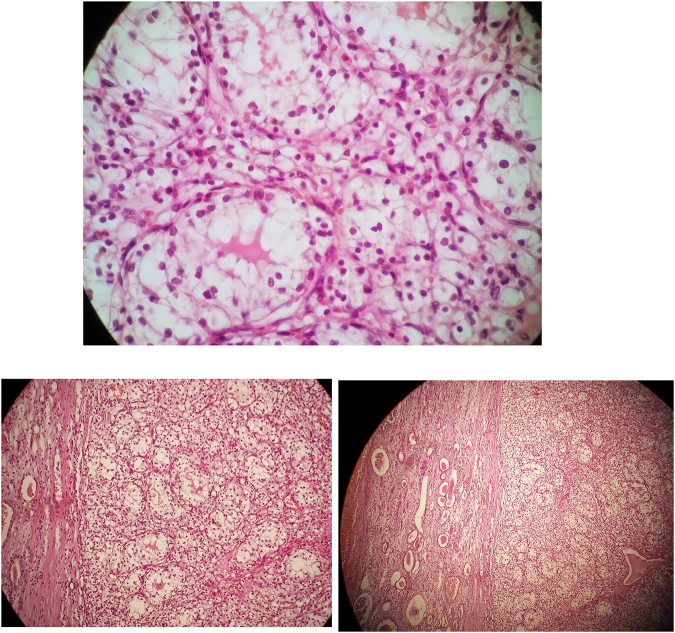
Microscopic images showing clear cell renal cell carcinoma grade I (hematoxylin and eosin staining).

**Figure 5.**
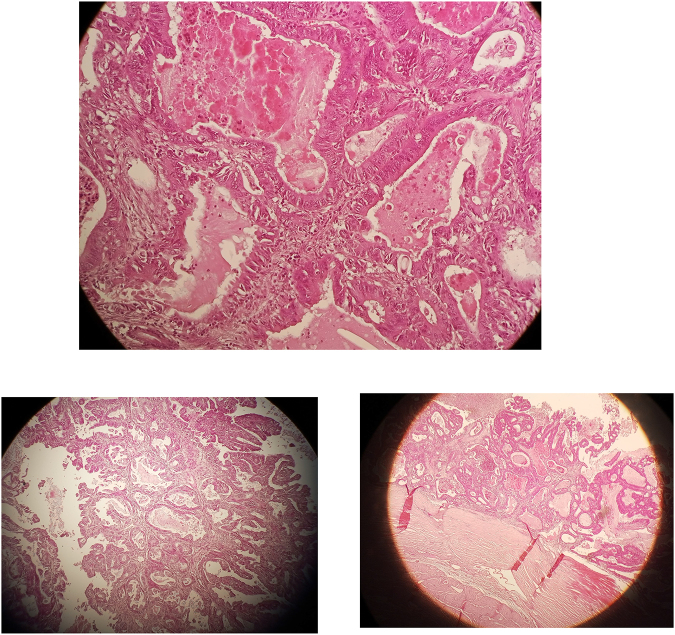
Figure shows microscopic images for papillary serous cystadenocarcinoma of the ovary grade II (hematoxylin and eosin staining).

Microscopically, hematoxylin and eosin-stained sections revealed grade I clear cell carcinoma confined to the kidney (Fig. 4) and grade II papillary serous cystadenocarcinoma extending beyond the right ovary surface (Fig. 5). The patient has been planned for adjuvant chemotherapy with six cycles of carboplatin and paclitaxel and was recommended for periodic follow-up; renal ultrasound and creatinine measurement were recommended every 3 months and CT scan of the abdomen and pelvis every 6 months.

## Discussion

Double primary tumors in one person are quite uncommon to coexist; only 1.84%–3.9% of all cancers have been observed to manifest with multiple malignancies in the same patient, with an incidence of 0.7%^[^5,6^]^. This is the first case to be reported in Syria as multiple primary tumors. The first person to describe MPMs was Billroth in 1889^[^6^]^. MPMs could be further classified into two categories based on the time interval between tumor diagnosis: synchronous malignancies are second tumors that occurred concurrently or within 6 months of the first malignancy, whereas metachronous malignancies are secondary tumors that have developed beyond or after 6 months of the initial malignancy^[^5^]^.

The most common sites of synchronous primary tumors are the genitourinary and gastrointestinal tracts, then the breast and the genitourinary tract, and the breast and the gastrointestinal tract^[^7^]^. Nevertheless, it is very uncommon for ovaries and kidneys to develop simultaneous primary neoplasms^[^2^]^. Our patient developed two synchronous primary tumors in the kidney and the ovary.

The first case of primary ovarian and kidney carcinoma occurring simultaneously was reported by Myoga *et al* in 1988 in Japan^[^5^]^.

To our best knowledge, only 11 cases similar to ours have been recorded in the medical literature using Pubmed using keywords (synchronous tumors, renal, and ovary) from 2000 till 2025^[^4,5,8,9^]^. Olaru^[^8^]^ documented a case involving a serous papillary cystadenocarcinoma in the right ovary followed by a clear cell renal cell carcinoma in the left kidney 3 months later; he also mentioned that only seven cases of synchronous primary ovarian cancer and renal cell carcinoma had been documented in the literature before his case. Duquesne *et al*^[^9^]^ presented a case of synchronous heart, ovaries, and kidney tumors in a 63-year-old Caucasian female patient who primarily attended their hospital for a hypertension evaluation. Waseem *et al*^[^5^]^ presented a case of a 60-year-old lady who presented with abdomen pain and her reports showed a right kidney mass and ovarian mass which suggested it to be a metastatic case but after surgery immunohistochemistry from the masses the patient was diagnosed with bilateral ovarian adenocarcinoma and a simultaneous clear cell carcinoma of the right kidney^[^5^]^.

Furthermore, Singh *et al*’*s*^[^4^]^ report in 2020 was the most recent one discussing primary synchronous tumors of the kidney and ovaries, detailing a case of clear cell carcinoma in the right kidney and papillary adenocarcinoma in both ovaries.

Renal cell carcinoma (RCC) is the most common type of kidney cancer and has several subtypes; clear-cell RCC is the most prevalent subtype, making up around 70% of cases^[^10^]^. On the other hand, ovarian cancer has various subtypes, with the most common being epithelial cancer, which accounts for 90% of cases. This subtype has five major histologies that differ in their origin, molecular profile, and clinical prognosis. Non-mucinous tumors make up 97% of epithelial ovarian cancer, while mucinous tumors have different histotypes such as serous 70%, endometrioid 10%, clear cell 10%, and unspecified 5%^[^11^]^. Our patient was diagnosed with left renal clear cell carcinoma grade I measuring 8 cm in its greatest dimension, confined to the kidney and right ovarian papillary serous cystadenocarcinoma grade II measuring 11 cm in its greatest dimension and extending beyond the ovary surface.

Multiple primary cancers can occur for a variety of reasons, but the most common ones are genetic susceptibility, patient immune systems, and extensive exposure to carcinogens such as chemotherapy and/or radiation therapy used to treat tumors; nevertheless, the exact causes of these mechanisms are still unknown^[^5,12^]^. Additionally, there have been few reports that point towards an association between RCC and steroid hormone target organs (breasts, uterus, and ovaries). In 2020, a study mentioned 17 cases of RCCs associated with second primary neoplasms occurring in steroid hormone target tissues. Among them, 10 had associated breast carcinoma, 4 had endometrial carcinoma and only three had ovarian carcinoma^[^4^]^. However, our patient didn’t mention any previous chemotherapy, radiotherapy, nor steroidal hormone intake. Concolino *et al* noticed that cytosol from normal human kidney specimens showed binding activity for steroid hormones, which was related to a receptor specific only for estradiol and progesterone. How estrogen induces tumorigenesis in the kidney remains to be clarified^[^5^]^.

Additionally, the differentiation between metastatic tumors and double primary tumors sometimes poses a diagnostic challenge. Observing distinctly diverse histologies is necessary to create a definitive diagnosis of coexisting double tumors. Warren and Gates reported in 1932 the following criteria for the diagnosis of double primary malignancies: (1) Histological proof that the secondary tumors and the index tumors are malignant. (2) Normal mucosa should be present between the tumors for at least 2 cm and there should be a minimum of 5 years between tumors if they are in the same region. (3) It is necessary to eliminate any chance of metastatic progression^[^5^]^.

Distinguishing between coexistent double cancers and metastasis can be challenging before surgery. Preoperative imaging studies such as CT or MRI typically show small, multiple, bilateral renal metastases located within the renal capsule, while primary RCC is usually single and unilateral, as demonstrated in our case^[^6^]^. Although diagnosing multiple tumors is challenging; however, the management of the patients with these tumors is also important. Doctors have to find a therapy suitable for both malignancies without increased toxicity, pharmacological interactions, or negative impact on the overall impact^[^4^]^. Our patient will receive six cycles of carboplatin and paclitaxel as adjuvant chemotherapy after she underwent radical nephrectomy and resection of the right ovary.

This work has been reported in line with the SCARE 2023 criteria^[^13^]^.

## Conclusion

In clinical practice, it’s important to consider the possibility of both ovarian cancer and RCC occurring together, despite its extreme rarity. Distinguishing between two separate primary tumors and metastasis requires a thorough examination of both pathological and radiological characteristics.

## Data Availability

All data can be made available upon request to the corresponding authors.

## References

[1] Zhai C Cai Y Lou F. Multiple primary malignant tumors—a clinical analysis of 15,321 patients with malignancies at a single center in China. J Cancer 2018;9:2795–801. 30123347 10.7150/jca.25482 PMC6096360

[2] Tsili AC Charisiadi A Koliopoulos G. Synchronous primary tumors of the kidney and the ovaries: imaging findings. J Radiol Case Rep 2008;2:2–8. 22470603 10.3941/jrcr.v2i5.71 PMC3303247

[3] Demandante CG Troyer DA Miles TP. Multiple primary malignant neoplasms: case report and a comprehensive review of the literature. Am J Clin Oncol 2003;26:79–83. 12576929 10.1097/00000421-200302000-00015

[4] Singh K Gupta D Tewari A. Synchronous primary tumors of kidney and bilateral ovaries-a diagnostic challenge: case report. Eur J Med Health Sci 2020;2:2–8.

[5] Waseem RM Kumar VS Nath PS. Synchronous primary tumors of the kidney and the ovaries: case report. Medico Res Chron 2019;6:200–04.

[6] Huang K-H Chung S-D Huang S-Y. Coexistence of ovarian cancer and renal cell carcinoma. J Formos Med Assoc 2007;106:S15–S19. 17493903 10.1016/s0929-6646(09)60360-0

[7] Balat O Kudelka AP Ro JY. Two synchronous primary tumors of the ovary and kidney: a case report. Eur J Gynaecol Oncol 1996;17:257–59. 8856298

[8] Olaru OG. Synchronous primary serous ovarian cancer and renal cell carcinoma. Int J Gynaecol Obstet 2014;126:282. 24910423 10.1016/j.ijgo.2014.04.003

[9] Duquesne I Sanchez-Salas R Zannis K. Concomitant heart, ovaries, and renal neoplasms: atypical findings during hypertension evaluation. Curr Urol Rep 2016;17:85. 27752942 10.1007/s11934-016-0645-8

[10] Díaz-Montero CM Rini BI Finke JH. The immunology of renal cell carcinoma. Nat Rev Nephrol 2020;16:721–35. 32733094 10.1038/s41581-020-0316-3

[11] Gaona-Luviano P Medina-Gaona LA Magaña-Pérez K. Epidemiology of ovarian cancer. Chin Clin Oncol 2020;9:47. 32648448 10.21037/cco-20-34

[12] Arpaci E Tokluoglu S Yetigyigit T. Multiple primary malignancies-a retrospective analysis at a single center in Turkey. Asian Pac J Cancer Prev 2013;14:769–73. 23621235 10.7314/apjcp.2013.14.2.769

[13] Sohrabi C Mathew G Maria N. The SCARE 2023 guideline: updating consensus Surgical CAse REport (SCARE) guidelines. Int J Surg Lond Engl 2023;109:1136. 10.1097/JS9.0000000000000373 PMC10389401 37013953

